# Notes and News

**Published:** 1888-08-18

**Authors:** 


### NOTES AND NEWS.

COLLECTED AND COMMUNICATED.

THIRTY thousand persons assembled, in bad weather, at Foxholes to celebrate the annual gala of the Rochdale Infirmary.

DUNDEE ROYAL INFIRMARY.—Miss Symers, of St. Helens, has contributed £1,000 to the endowment fund of the Convalescent Home, Barnhill, in connexion with the Dundee Royal Infirmary.

AT a meeting of the governors of Anderson's College Medical School, Glasgow, held on the 3rd inst., Dr. Alexander Napier, Crosshill, was elected Professor of Materia Medica in room of Dr. Morton resigned.

BY his will, the late Mr. David Buchan, who resided at Tor, Murrayfield, Edinburgh, has bequeathed the following legacies to Edinburgh charitable institutions:—The Edinburgh Royal infirmary, £10,000; the Convalescent Home, near Corstorphine, £2,000; the Royal Edinburgh Hospital for Sick Children, £200; the Society in Edinburgh for the Relief of the Destitute Sick, £200; and the Edinburgh Hospital for Incurables, £200.

VALUABLE presentations were made last week at a Quarterly Board of Governors to three honoured officials of the Oldham Infirmary. To Mr. George Lees, a former secretary, there was presented a threequarter-plate keyless English lever watch, jewelled in rubies, with heavy gold case; to Mr. G. Wainwright, J.P., the treasurer, silver candelabra and candlesticks, of Corinthian pattern; and to Dr. McGowan, a handsome marble clock, with side ornaments. These little amenities of hospital life show a mutual regard and esteem which it is exceedingly pleasant to read about.

AMONG recent naval appointments is that of Surgeon Edward A. Spiller to the *Albacore*, and Surgeon T. A. Levinge to the *Tronti*.

MR. EDWARD ST. AUBYN, Mr. C. Wake, and Mr. C. A Shepcote, the chairman of committee, hon. treasurer, and hon. secretary respectively of the Royal Albert Asylum, Devonport, are now appealing for funds to enable them to carry on the work of reformation they have undertaken with regard to the patients in the lock wards of the hospital. Much good has been done by the labours of the chaplain and the matron, assisted by several other ladies, in this field, and it would be matter for the deepest regret if it had to be curtailed for lack of the necessary money, which is now rather likely, as the subsidy received from the Admiralty no longer provides any sum that can be used for this purpose.

OWING to the fact that the Bristol Docks Company intend to pay back £30,000 invested with them twenty-one years ago by the authorities of the Bristol Infirmary, and that no investment can be found for the money which will give such high interest, the infirmary is threatened with a serious diminution of income. It has finally been decided to invest the money in Bristol Corporation Bonds, but these will give only 3½ per cent., instead of the 4½ given by the docks, and must be purchased at the rate of 108 per cent. This means a loss to the institution of nearly £400 a-year, but it is apparently the best investment open to the trustees.

IN the Aerztekammer, or medical parliament, recently established in Prussia, the question of homes for women recently discharged from lying-in hospitals is being discussed. When an institution takes on itself the responsibility of receiving poor women and affording them medical attendance during childbirth, it necessarily follows that that responsibility does not cease directly the patient is discharged. Long after recovery from childbirth the patient is quite unfit to pursue avocations of that arduous kind which women of the poorer classes are called upon to perform. Hard work, insufficient food, and unhealthy surroundings, expose her to risks of permanent discomfort, well-known to all practitioners and obstetricians. These truths are recognised by the medical authorities in the Prussian capital, and the Berlin Gynæcological Society is engaged in preparing a scheme which Dr. Martin will report to the Aerztekammer. The working of convalescent lying-in hospitals in the United Kingdom will be carefully investigated.

THE following vacancies are announced this week, the amount of salary in each case being given after the name of the institution :—

**Resident Medical Officers.**

Belgrave Hospital for Children. Resident House Surgeon and Assistant Secretary.—£30 per annum, with board, etc.
City of Liverpool Infectious Diseases Hospital. Resident Medical Officer.—£100 per annum, with board, etc.
General Hospital, Birmingham. Assistant House Surgeon.—Board; no salary.
Liverpool Dispensaries. Assistant Surgeon.—£80, with board, etc.
Royal Berks Hospital, Reading. Assistant House Surgeon, unqualified.—Board; no salary.
Royal Hospital of Bethlem. Assistant Medical Officer.—£300 per annum, with furnished residence. Candidates must be M. or F.R.C.S. and M. or L.R.C.P., or L.S.A.
Swansea Hospital. Resident Medical Officer.—£100 per annum, with board, etc.
Victoria Hospital, Burnley. Resident Medical Officer.—£60.

**Non-resident Medical Officers.**

Hulme Dispensary, Manchester. Honorary Surgeon.
Ital an Hospital, Queen Square, Bloomsbury. Assistant Medical Officer.
Samaritan Free Hospital for Women and Children. Physician, out department.
Swinford Union, Low Park Dispensary. Medical Officer.—£120 per annum, and fees.
Universi'y College, Bristol. Medical Tutor.—£125 per annum.
University of Aberdeen. Chair of Chemistry.
Wilton Union. Medical Officer and Public Vaccinator for Stapleford District.

---

HOLIDAY-MAKING seems to be more popular than ever. It is stated that about 1,600 persons cross to the Continent daily from Folkestone and Dover. Foreigners are also said to be finding their way to this country in unprecedented numbers.

---

DR. FARQUHARSON, M.P., addressing the members of the British Medical Association at Glasgow, said: "There was no doubt that scarlet fever and other diseases, such as anthrax, could be transmitted from animals to man, but all those evils fell into insignificance before that terrible tuberculosis which, after what they had heard, was evidently also capable of being transmitted from animals to man. They should not rest till they had this disease of tuberculosis included among those other diseases of animals dealt with in the Contagious Diseases (Animals) Act."

---

PRESIDENT GAIRDNER, addressing the members of the British Medical Association in session at Glasgow, said that in Charles Darwin, when his character was carefully studied, they had a man of the very stuff and moral fibre of which the most eminent saints were made. Dr. Gairdner also expressed the emphatic conviction that the physician of the future would not be less but more inclined to make a study of his Bible than heretofore. But he would study it in the spirit of moderation, scientific freedom, and historical research, and not under the influence of mere tradition and ecclesiastical authority. It was thus only, it seemed to him, that the reconciliation of science and religion could be brought about.

---

A QUARTERLY court of the Governors of the Consumption Hospital, Brompton, was held at the hospital on the 6th inst., Mr. T. P. Beckwith in the chair. The report of the committee of management, read by the secretary (Mr. Dobbin) stated that since the alterations the whole of the beds had become reoccupied, and the work of the charity had been carried on at its highest point of usefulness. The plan of sending patients to convalescent homes at the seaside at the expense of the hospital (which was adopted during the alterations as a temporary expedient), having proved so beneficial, the committee proposed to continue the arrangement provisionally, within certain limitations. By the receipt of the reversionary bequest of the late Mr. George Stone (which had been long expected), a very acceptable addition had been made to the reserve fund, upon which it had been found necessary to draw heavily from time to time to meet current expenditure. The following legacies had been announced since the last court: Sir Robert Loder, Bart., £2,500; Mr. D. Milner, moiety of residue, contingent; Mrs. Annie Kirkup, £500, reversionary; Lady Buchan, £500; Miss G. Austin, £500, duty free. A donation of 100 guineas had been received from Mr. John Wilson Theobald, in order to name a memorial bed. The number of in-patients admitted since May 31st was 239; discharged, many greatly benefited, 262; died, 39; new out-patient cases, 2,155.

---

THE fifty-sixth annual meeting of the British Medical Association, in Glasgow, has now come to a successful close. The first general meeting for the transaction of business was held on the morning of August 7th; in the afternoon a service was held in St. Mungo's Cathedral, at which a sermon was preached by the Very Rev. John Caird, D.D., Principal and Vice-Chancellor of the University, and in the evening an address was delivered by the president-elect of the Association, Dr. W. T. Gairdner, professor of medicine in the University, and physician to the Queen in Scotland, dealing especially with certain aspects of modern education. On the following day the scientific work of the meeting, which is conducted by twelve sections, was commenced, and in the afternoon Dr. Clifford Allbutt, F.R.S., of Leeds, gave an address in medicine on the subject of Comparative Nosology. On August 9th and 10th the sectional meetings were continued, and on the first-named day two addresses were delivered on surgical subjects by two eminent Glasgow surgeons, Sir George H. B. Macleod, Regius Professor of Surgery in the University, and Surgeon to the Queen in Scotland, and Dr. William Macewen. The concluding general meeting was held on August 10th. Dr. J. G. McKendrick, F.R.S., Professor ef the Institutes of Medicine in the University, gave an address on the "Chemistry of the Blood." The public dinner of the association was held in St. Andrew's Hall on August 9th, and among other entertainments were a conversazione by the Principal and Professors of the University, a garden party given by the faculty of Physicians and Surgeons in the Botanic Gardens, and a conversazione given by the Corporation of Glasgow.

---

THE Lewes Dispensary, which has been altered and extended in commemoration of her Majesty's Jubilee, was re-opened on the 3rd inst., by the Mayor (Alderman Farncombe), in the presence of a large assembly of ladies and gentlemen. In addition to his Worship there were present at the ceremony: The Mayoress (Mrs. Farncombe), Viscount Hampden and Lady Hampden, the Rev. Lord Sydney Godolphin Osborne, Mr. R. Crosskey (chairman of directors), Alderman Crosskey, M.D., Mr. H. Card (the architect), and others interested in the institution. The Rev. Lord Sydney Godolphin Osborne delivered the special dedicatory prayer. Mr. R. Crosskey said that the resolution of the inhabitants of Lewes to commemorate her Majesty's Jubilee by altering and extending the dispensary had enabled the directors to effect the improvement in the building that they saw that day. The additions and alterations were well planned by their architect, the borough surveyor, Mr. Card, and had been ably carried out by the contractor, Mr. Floyd. Viscount Hampden, who was received with applause, said, among other remarks, that among the various objects which had been undertaken on the occasion of the Queen's Jubilee, he knew none more appropriate that that of relieving the sufferings of humanity. The Mayor said it was his privilege and pleasure to hand to the secretary of the institution a cheque for some £600 or £700, part of the subscriptions raised for the celebration of the Jubilee last year. The secretary had informed him that the cost of the extension was about £1,000—a sum somewhat larger than the amount he received to the Jubilee fund; but it was gratifying to know that the bulk of the money was raised by the Jubilee subscriptions of the last year.

